# Supplementary material for: 7,8-Dihydroxyflavone is a direct inhibitor of human and murine pyridoxal phosphatase
Source: eLife. 2024 Jun 10;13:RP93094. doi: 10.7554/eLife.93094 (PMC11164532; doi:10.7554/eLife.93094)
Supplement: Figure 1—source data 3. — Each value represents the result of the PLP determination in an individual hippocampus. Analysis for statistically significant differences between PLP levels in PDXP-WT and PDXP-KO hippocampi (all ages combined; two-tailed, unpaired t-test) p<0.0001. Bold table entries indicate those hippocampal extracts that were further separated for an analysis of protein-depleted and protein-bound PLP (see Figure 1c). Source data are available for this table. [file elife-93094-fig1-data3.zip › Brenner_Figure_1figure_supplement_1.docx]

| PDXP-WT | |  | PDXP-KO | |
| --- | --- | --- | --- | --- |
| age [days] | PLP  [nmol/g protein] |  | age  [days] | PLP  [nmol/g protein] |
| 18 | 66.77 |  | 18 | **154.60** |
| 18 | **111.66** |  | 18 | **144.50** |
| 18 | **110.11** |  | 18 | **154.74** |
| 39 | **99.73** |  | 34 | **169.06** |
| 39 | 107.59 |  | 34 | **177.57** |
| 39 | 109.60 |  | 40 | 153.93 |
| 42 | 89.62 |  | 41 | **180.65** |
| 42 | **105.82** |  | 41 | 157.84 |
| 42 | **104.38** |  | 41 | 166.67 |
| 59 | 71.55 |  | 60 | 180.58 |
| 59 | 68.99 |  | 60 | 142.33 |
| 84 | 59.36 |  | 87 | 214.52 |
| 84 | 65.52 |  | 87 | 198.61 |
| 86 | 73.50 |  | 94 | 196.21 |
| 86 | 74.40 |  | 94 | 211.20 |
| 94 | 123.81 |  | 94 | 214.70 |
| 94 | 116.81 |  | 94 | 201.21 |
| 94 | 138.95 |  | 94 | 230.42 |
| 136 | 84.61 |  | 142 | 192.13 |
| 176 | 72.22 |  | 142 | 188.03 |
| 176 | 72.83 |  | 162 | 143.41 |
| 252 | **83.47** |  | 162 | 158.23 |
| 252 | **85.78** |  | 256 | **180.26** |
| 252 | **78.88** |  | 256 | **184.36** |
| 252 | 72.86 |  | 256 | **168.17** |
| 252 | 62.20 |  | 256 | 191.69 |
| 252 | 69.15 |  | 256 | 182.87 |
| 269 | 72.53 |  | 256 | 189.11 |
| 269 | 90.90 |  | 327 | 149.04 |
| 269 | 80.60 |  | 327 | 139.20 |
| 335 | 52.90 |  | 351 | 165.84 |
| 335 | **60.93** |  | 351 | **163.33** |
| 335 | **52.17** |  | 351 | **142.23** |
| 335 | **65.93** |  | 351 | **155.71** |
|  |  |  | 366 | 156.26 |
