# Supplementary material for: 7,8-Dihydroxyflavone is a direct inhibitor of human and murine pyridoxal phosphatase
Source: eLife. 2024 Jun 10;13:RP93094. doi: 10.7554/eLife.93094 (PMC11164532; doi:10.7554/eLife.93094)
Supplement: Figure 2—source data 5. — Determination of half-maximal inhibitory constants (IC50) of 11 PDXP inhibitory compounds (see InChI Key for chemical substance identification) using purified murine PDXP and pyridoxal 5’-phosphate (PLP) as a substrate. Data marked with an asterisk (*) are results of n=3 biologically independent experiments. Because of the limited quantity of most compounds available for these assays, all other data are results of n=1 determinations. 7,8-DHF, 7,8-dihydroxyflavone. [file elife-93094-fig2-data5.zip › Brenner_Figure_2figure_supplement_2.docx]

|  | **InChI Key** | **IC_50_ [µM]** |
| --- | --- | --- |
| 7,8-DHF | COCYGNDCWFKTMF-UHFFFAOYSA-N | 0.8 * |
|  | RDMYXZLESVAHOO-UHFFFAOYSA-N | >40 |
|  | RBZAGLIUHTVMFL-UHFFFAOYSA-N | >40 |
|  | HSJXOMZEPTVVQC-UHFFFAOYSA-N | >40 |
|  | PRAORRBPQXAGQD-UHFFFAOYSA-N | >40 |
|  | BMXOLQCTPIQVOD-UHFFFAOYSA-N | >40 |
|  | GYJACFGQPFXKCA-UHFFFAOYSA-N | >40 |
|  | PCRFOVGMSDFVLC-UHFFFAOYSA-N | >40 |
|  | FHXDSQYFCFWKAW-UHFFFAOYSA-N | >40 |
|  | OPTDAYWBFJRIGB-UHFFFAOYSA-N | >40 |
|  | GCUCIFQCGJIRNT-UHFFFAOYSA-N | >40 |
